# Testicular Melatonin and Its Pathway in Roe Deer Bucks (*Capreolus capreolus*) during Pre- and Post-Rut Periods: Correlation with Testicular Involution

**DOI:** 10.3390/ani11071874

**Published:** 2021-06-23

**Authors:** Alberto Elmi, Nadia Govoni, Augusta Zannoni, Martina Bertocchi, Chiara Bernardini, Monica Forni, Domenico Ventrella, Maria Laura Bacci

**Affiliations:** Department of Veterinary Medical Sciences, University of Bologna, 40064 Ozzano Dell’Emilia, Italy; alberto.elmi2@unibo.it (A.E.); nadia.govoni@unibo.it (N.G.); augusta.zannoni@unibo.it (A.Z.); martina.bertocchi3@unibo.it (M.B.); chiara.bernardini5@unibo.it (C.B.); monica.forni@unibo.it (M.F.); marialaura.bacci@unibo.it (M.L.B.)

**Keywords:** *Capreolus capreolus*, roe deer, testicular cycle, melatonin, ANAAT, ASMT, melatonin receptors, reproductive physiology, seasonal breeder

## Abstract

**Simple Summary:**

The roe deer is a small wild ruminant, very common in Europe and Asia; adult specimens are sexually active only during summer, in very short timeframes. Peculiarly, males, also known as bucks, produce spermatozoa only in this period, with a subsequent morph-functional testicular involution. In seasonal breeders, melatonin plays a pivotal role by converting light information and controlling the testicular hormonal function and, recently, its local production within testes has been described in other species. The aim of the present work was to study testicular melatonin and its synthesis pathway in roe deer during the pre-rut (June–July) and post-rut (August–September) periods, and correlate it with morph-functional testicular changes. Samples were opportunistically obtained from hunted specimens according to the local hunting calendar. The results also seem to suggest a local melatonin production in this species, but no correlations with testicular involution parameters were highlighted, probably due to the very short sampling timeframe. More studies are necessary to understand the role of melatonin in the testicular cycle and provide more information regarding the interesting reproductive physiology of this species.

**Abstract:**

Roe deer are seasonal breeders with a complete yearly testicular cycle. The peak in reproductive activity is recorded during summer, the rutting period, with the highest levels of androgens and testicular weight. Melatonin plays a pivotal role in seasonal breeders by stimulating the hypothalamus–pituitary–gonads axis and acting locally; in different species, its synthesis within testes has been reported. The aim of this study was to evaluate the physiological melatonin pattern within roe deer testes by comparing data obtained from animals sampled during pre- and post-rut periods. Melatonin was quantified in testicular parenchyma, along with the genetic expression of enzymes involved in its local synthesis (AANAT and ASMT) and function (UCP1). Melatonin receptors, MT1-2, were quantified both at protein and gene expression levels. Finally, to assess changes in reproductive hormonal profiles, testicular dehydroepiandrosterone (DHEA) was quantified and used for a correlation analysis. Melatonin and AANAT were detected in all samples, without significant differences between pre- and post-rut periods. Despite DHEA levels confirming testicular involution during the post-rut period, no correlations appeared between such involution and melatonin pathways. This study represents the first report regarding melatonin synthesis in roe deer testes, opening the way for future prospective studies in the physiology of this species.

## 1. Introduction

Seasonal variations in reproductive activity represent the main adaptive response to climatic changes in temperature and food availability in wild species, leading to parturitions only during the most favorable environmental periods [1]. This phenomenon is characterized, in many species, by seasonal gonadal cycles, well described in amphibians [2], reptiles [3], birds [4,5] and, amongst mammals, in hamsters [6], moles [7], visons [8], and bison [9].

Generally, the seasonal timing of the reproductive cycle is controlled by melatonin, a protein hormone derived from tryptophan and mainly produced within the pineal gland in response to darkness [10]. Melatonin is only released during night hours, and for a longer time span, and often higher levels, in winter compared to summer [11]. It serves as a neurotransmitter, passing photic information from the retina to the hypothalamic–pituitary–gonadal (HPG) axis, contributing to the modulation of gonadotropin hormone release via GnRH (gonadotropin-releasing hormone) and GnIH (gonadotropin-inhibiting hormone) [10,12]. The nature of the feedback (release or inhibition) evoked in the HPG axis depends on the species-specific characteristics of the reproductive cycle: in “long days seasonal breeders” (e.g., hamsters and horses), melatonin is interpreted as an anti-gonadotropic event; indeed, reproduction takes place in spring/summer when daylight hours are longer. On the other hand, in “short day seasonal breeders” (e.g., goat and sheep), it is considered pro-gonadotropic, with the sexual activity peaking during winter [13,14]. Finally, in non-seasonal breeders such as humans, pigs and bovine, where reproduction takes place throughout the entire year, the role of melatonin is stilly poorly understood [12,13].

In addition to its modulator activity on the HPG axis, melatonin is capable of passing through the blood–testis barrier, exerting a direct action on testicular tissue by binding its specific receptors 1 and 2 (MT1 and MT2) and regulating androgen production, increasing Sertoli cells responsiveness to FSH during testicular development, and modulating cellular growth and proliferation [12,15]. MT1 and MT2 are G protein-coupled receptors present in both Leydig and Sertoli cells in many animal species [10,16,17,18,19,20]. In addition, melatonin can also be directly synthesized within the testis upon the action of two pivotal enzymes: aralkylamine-N-acetyltransferase (AANAT) and N-acetylserotonin-O-methyltransferase (ASMT), which catalyze the conversion of serotonin (5-HT) into melatonin [21,22,23,24].

Among non-domestic mammals, the *cervidae* family shows a very marked annual reproductive cycle [1]: roe deer bucks (*Capreolus capreolus*, Linneus 1758), in particular, are characterized by an extremely interesting testicular cycle. Sexually mature male show seasonal alternation between morpho-functional involution (summer end/autumn) and recrudescence (spring), with transitions between totally arrested (winter) and highly active spermatogenesis (spring/summer). When looking at the reproductive season, three main well-timed periods can be identified: pre-rut (June/mid-July), a short rutting window synchronized with the female oestrus (mid-July/mid-August), and post-rut (mid-August/September) [25,26]. Starting from the end of the rutting period, testes show a morpho-functional involution characterized by a reduction in volume (up to 20%), a reduction in sexual hormones’ levels, and a decline of the germ cell line, although without apoptotic events [27,28,29]. Despite several aspects and biomarkers having been investigated, including hormones (FSH, LH, prolactin, estrogens) [26,30,31,32], androgen receptor distributions [33], growth factors (TGF, IGF, FGF, VEGF) [34], gelatinases (proMMP2) [35], and antioxidant enzymes (superoxide dismutase, glutathione peroxidase, catalase) [36], the processes underlying the annual testicular cycle are still not fully clear. Koziol and colleagues [37] recently reported melatonin concentrations in peripheral blood and the presence of melatonin receptors (MT1 and MT2) in the testis and epididymis of roe deer sampled during different reproductive periods. Plasmatic melatonin was higher in the pre- and post-rut periods when compared to the rutting period, whereas MT1 and MT2 expression levels were lower starting from September in both tissues. Unfortunately, no data were reported regarding testicular melatonin. Therefore, melatonin seems to be involved in the regulation of the male roe deer reproductive cycle, potentially by promoting the proliferation and development of germ cells, although its accurate local and systemic mechanisms of action are still to be clarified [37].

The aim of the present study was to evaluate the physiological pattern of melatonin within roe deer testes by comparing data obtained from animals sampled during pre- and post-rut periods. In particular, melatonin concentrations in testicular parenchyma were quantified, along with the genetic expression of enzymes involved in its local synthesis (AANAT and ASMT) and, potentially, functioning as the uncoupling protein 1 (UCP1, involved in energy release by heat dissipation). Expression of melatonin receptors MT1 and MT2 was quantified both at protein and messenger RNA levels. Finally, in order to assess changes in the reproductive hormonal profile, the testicular levels of dehydroepiandrosterone (DHEA), an androgen adrenal testosterone prohormone capable of non-sexually related functions [38,39], were quantified and used for a correlation analysis implemented with previously reported data of the same animals [35], such as testis weight and testosterone (T).

## 2. Materials and Methods

### 2.1. Animals

Samples from 18 roe deer bucks (*n* = 18) hunted in 2018, as per the local hunting calendar (Resolution No. 792/2018 of the Emilia Romagna Regional Executive) in the South-Western Bologna Apennines (Italy), were used for the present study. Half of the animals were culled during pre-rut (1 June–15 July; *n* = 9), while the other half were culled during post-rut (15 August–30 September; *n* = 9). Environment variables, including hours of daylight and min/max temperatures, were recorded. All the biological specimens analyzed in this study were obtained from hunted animals (in accordance with the hunting plan in force); no ethical approval was necessary.

All animals included in the study were considered healthy based on gross examination. Upon death, the scrota with both testes were immediately collected by the personnel of the local biometrical center and refrigerated at 5 ± 1 °C. Within two hours, all samples were moved to the physiology laboratories of Department of Veterinary Medical Sciences of the University of Bologna (Ozzano dell’Emilia, Italy). Ages were assessed upon an analysis of teeth eruption and wear patterns as previously described [35].

### 2.2. Testicular Tissue Sampling

Testicular parenchyma was collected and stored as previously described [35]. Briefly, upon testis isolation, tissue was collected from the middle section, minced, and split into 3 aliquots: two were snap-frozen in liquid nitrogen and stored at −80 °C for hormonal quantification and Western blot analysis; the other one was incubated for one day with an RNA stabilization solution (RNAlater™, Thermo Fisher Scientific, Watham, MA, USA) and stored at −80 °C after solution removal, for gene expression analyses.

### 2.3. Melatonin Quantification in Testicular Parenchyma

Melatonin concentrations in roe deer testes were measured by means of a commercial competitive immunoassay kit (Melatonin Elisa kit, IBL international, Hamburg, Germany) following the manufacturer’s instructions. Testes samples were homogenized for 1 min in PBS with 0.1% ethanol at a final tissue concentration of 140 mg/mL, sonicated on ice for 30 s, and then centrifuged at 3000× *g* for 20 min ([17], partially modified). Standards (range 3–290 pg/mL), controls, and testicular homogenates were extracted using C18 extraction columns (IBL, Hamburg, Germany) according to the manufacturer’s protocol (the yield of extraction with this procedure is approx. 90–100%). The extracts in methanol were evaporated to dryness under an air-stream suction hood. The dried extracts were then reconstituted with 0.15 mL of bidistilled water and assayed immediately. Briefly, 50 µL of each extracted standard, control and sample was loaded in duplicate in a microtiter plate coated with anti-rabbit IgG; then, 50 µL of biotinylated melatonin and 50 µL of melatonin antiserum were added to each well and incubate for 14–20 h at 2–8 °C. After three washes, 150 µL of enzyme conjugate was loaded to the wells and incubated for a further 120 min on an orbital shaker set at 500 rpm. Wells were washed three times again, and 200 µL of substrate solution was added to each well, which were incubated for 40 min on a plate rotator at 500 rpm. After incubation, 50 µL of stop solution was added and absorbance was measured using a microplate reader at 405 nm (Infinite F50 Tecan, Grodig, Austria). The cross-reactivity values were: 5-methoxy-tryptophole (1.2%), N-acetyl-serotonin (1.2%), 5-methoxy-tryptamine (2.5%), and other substances tested <0.01%. The analytical sensitivity was 2.6 pg/well and intra-assay variability was 5.3%. Linearity was evaluated with pooled samples serially diluted (1:1–1:8) in the buffer kit. A good parallelism between sample dilution and the standard curve was found (R^2^ = 0.99). Melatonin concentrations in testes were expressed as pg/mg tissue weight.

### 2.4. DHEA Quantification in Testicular Parenchyma

Steroid extraction from testis samples was performed as previously described [35]. Briefly, samples were homogenized in PBS to prepare 10% (*w*/*v*) homogenate and centrifuged at 3000× *g* for 10 min. Steroids were extracted overnight, following our internal standard procedure, by adding 0.2 mL of homogenate to 5 mL of methanol. After centrifugation, 4 mL of supernatant was placed in a glass tube, evaporated, and stored until analysis. Extracts were reconstituted in assay buffer (1 mL), and 0.1 mL was used for measurements of dehydroepiandrosterone (DHEA) by radioimmunoassay; tritiated DHEA (30 pg/tube; 76.1 Ci/mmol; PerkinElmer inc. Boston, MA, USA) was added, followed by rabbit anti-DHEA serum (0.1 mL, 1:10,000; produced in our laboratory). After the incubation and separation of antibody-bound and -unbound steroids by charcoal–dextran solution (charcoal 0.25%, dextran 0.02% in phosphate buffer), tubes were centrifuged (15 min, 3000× *g*), the supernatant was decanted, and radioactivity was immediately measured using a β-scintillation counter (Packard C1600, Perkin Elmer, Waltham, MA, USA). The sensitivity of the assay was 4.6 pg/tube, and the precision within tests was assessed by calculating intra-assay coefficients of variation from all duplicated samples analyzed, which was 4.9%. Cross reactions of various steroids with antiserum raised against DHEA were: DHEA (100%), DHEA sulfate (39%), androstenedione (1%), testosterone (0.25%), progesterone (0.01%), and cortisol (0.001%), as previously reported [39].

Parallelism between standards and endogenous hormones were determined by serially diluting (1:1–1:8) a pooled sample showing high DHEA levels with assay buffer. A regression analysis was used to determine parallelism between the two hormone levels in the same assay. A high degree of parallelism was confirmed by regression tests (R^2^ = 0.99). The assay results were expressed as pg/mg of tissue.

### 2.5. Western Blot for MT1 and MT2

Fifty milligrams of tissue was homogenized in 500 µL of SDS buffer (62.5 mM Tris-HCl pH 6.8; 2% SDS; 5% glycerol) supplemented with a protease inhibitor cocktail (Sigma-Aldrich, Co, St. Louis, MO, USA). Total protein content was determined by Peterson’s Modification of Lowry Method using a Protein Assay Kit (Sigma-Aldrich, Co, St. Louis, MO, USA). The quality of protein content was checked by SDS-Page electrophoresis followed by a Coomassie Blue staining-based method. After that, 20 μg of total proteins was separated on NuPage 4–12% bis-Tris Gel (Life Technologies Ltd., Paisley, UK) for 45 min at 200 V. Proteins were then electrophoretically transferred onto a nitrocellulose membrane (Trans-Blot Turbo, Transfer System, Bio-Rad) and protein transfer was checked by staining with 0.2% Ponceau S. After blocking the nonspecific binding with 5% nonfat milk in PBS-T20 (PBS-0.1% Tween-20) at room temperature for 1 h, membranes were incubated with a 1:400 dilution of Anti-Melatonin Receptor 1 (ab87639, Abcam, Cambridge, CB2 0AX, UK) and with a 1:500 dilution of Anti-Melatonin Receptor 2 (ab203346 Abcam, Cambridge, CB2 0AX, UK) in PBS-T20. After several washings with PBS-T20, membranes were incubated with the appropriate secondary biotin-conjugate antibodies (1:100,000 dilution in PBS-T20, 1 h at RT) and then with a 1:1000 dilution of an anti-biotin horseradish peroxidase (HRP)-linked antibody (1 h at RT). The Western blots were developed using a chemiluminescent substrate Clarity Western Substrate, (Bio-Rad Laboratories Inc., Hercules, CA, USA) according to the manufacturer’s instructions. The intensities of the luminescent signal of the resultant bands and their molecular weight were calculated by the ChemiDoc Instrument using Lab Image Software (Bio-Rad). In order to normalize the MT1 and MT2 data on the housekeeping protein, membranes were stripped (washed for 5 min in water, then 5 min in 0.2 M NaOH, and then washed again in water) and re-probed for housekeeping α-tubulin (MA1-19162, Thermo Fisher Scientific). The relative protein contents (MT1 or MT2/α-tubulin) were expressed as arbitrary units (AUs).

### 2.6. RNA Extraction and qPCR for AANAT, ASMT, UCP1 and MT1-2

In order to extract RNA, TRI Reagent (Molecular Research Center Inc., Cincinnati, OH, USA) and NucleoSpin RNA II (Macherey-Nagel GmbH & Co. KG, Düren, Germany) kits were used as previously described [35]. RNA was spectrophotometrically (Denovix Inc. Wilmington, DE, USA) quantified (A260 nm), and its quality was assessed by gel electrophoresis on 1% agarose. An iScript cDNA Synthesis Kit (Bio-Rad Laboratories Inc., Hercules, CA, USA) was used to reverse-transcribe total RNA to cDNA. Gene expression profiles were evaluated by means of quantitative real-time PCR (qPCR) in a CFX96 thermal cycler (Bio-Rad), with SYBR Green detection for target genes (AANAT: aralkylamine N-acetyltransferase, ASMT: acetylserotonin O-methyltransferase, UCP1: uncoupling protein 1, MT1: melatonin receptor 1, MT2: melatonin receptor 2, all based on *Bos Taurus* sequences) and for reference genes (GAPDH: glyceraldehyde-3-phosphate dehydrogenase, based on roe deer sequence; HPRT1: hypoxanthine phosphoribosyltransferase 1, ACTb: beta-actin and B2M: beta-2-microglobulin, based on *Bos Taurus* sequences). Primers were either designed using Beacon Designer 2.07 (Premier Biosoft International, Palo Alto, CA, USA), as reported in Table 1, or purchased from QIAGEN (Hilden, Germany, RT2 qPCR Primer Assay for HPRT1; ACTb; B2M, Cat. No. PPB00330A; PPB00173A; PPS00031A, respectively). The amplification reactions were performed, as previously described [35], with SYBR Green Supermix (Bio-RAD). The real-time program included an initial denaturation period of 1.5 min at 95 °C, 40 cycles at 95 °C for 15 s, and 60 °C for 30 s, followed by a melting step with ramping from 55 °C to 95 °C at a rate of 0.5 °C/10 s. In addition to roe deer samples, RNA extraction and qPCR were also performed, when needed, on a bovine testis as positive control. The specificity of the amplified PCR products was confirmed by agarose gel electrophoresis and melting curve analysis. The relative expressions of the studied genes were normalized based on the geometric mean of the three reference genes.

The relative mRNA expressions of tested genes were evaluated by using the ∆Ct (∆ threshold cycle) method (∆ = Ct mean reference genes–Ct interest gene), which was directly correlated with the expression level. The data are reported as the ∆Ct mean ± SD for both groups.

### 2.7. Statistical Analysis

R 3.0.3 (the r Foundation for Statistical Computing) was used to perform the statistical evaluations; graphic representations were obtained using the software GraphPad Prism v.8 (GraphPad Software Inc., San Diego, CA, USA).

Descriptive statistics were calculated and reported as the mean and standard deviation (SD). Normal distributions were evaluated by means of Shapiro–Wilk tests, while the equality of variances in the two groups was assessed by means of Levene’s test: according to the results, hypothesis statistic parametric or non-parametric tests were performed.

Data regarding testicular weight and testosterone quantification, previously reported by the authors [35], were used for non-parametric Spearman’s rank test correlation analysis. Statistical significance was set at 95% C.I.

## 3. Results

All analyzed samples were collected from sexually mature adult bucks with ages ranging from 15 to 72 months and mean weights of 22.7 kg; no statistical differences were recorded regarding the aforementioned parameters between pre- and post-rut animals. The descriptive analysis of all the investigated parameters, including environmental factors, is reported in Table 1.

### 3.1. Melatonin and DHEA Quantification

**Figure 1:**
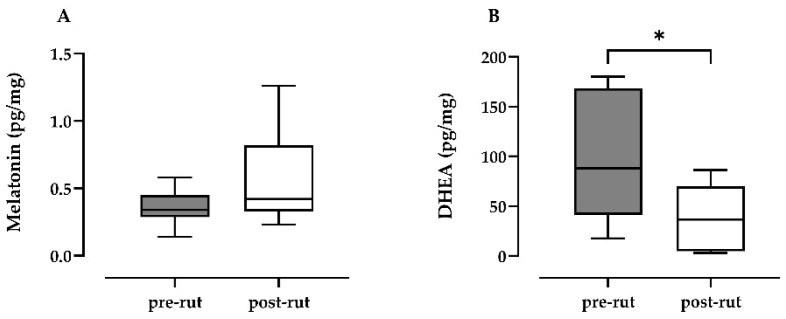
Differences between pre-and post-rut groups for the quantification of testicular parenchymal melatonin (**A**) and DHEA (**B**). *, *p* < 0.05. DHEA, dehydroepiandrosterone.

The authors [35] have previously reported the testicular involution (levels of testicular testosterone and testicular weight) between pre- and post-rut periods. The results of melatonin quantification in testicular parenchyma (Figure 1A) showed increased levels in the post-rut period, although this was not statistically significant (*p* = 0.3213). On the other hand, DHEA (Figure 1B) was statistically lower in the post-rut period (*p* = 0.0360).

### 3.2. qPCR for AANAT, ASMT, UCP1, MT1-2

**Figure 2:**
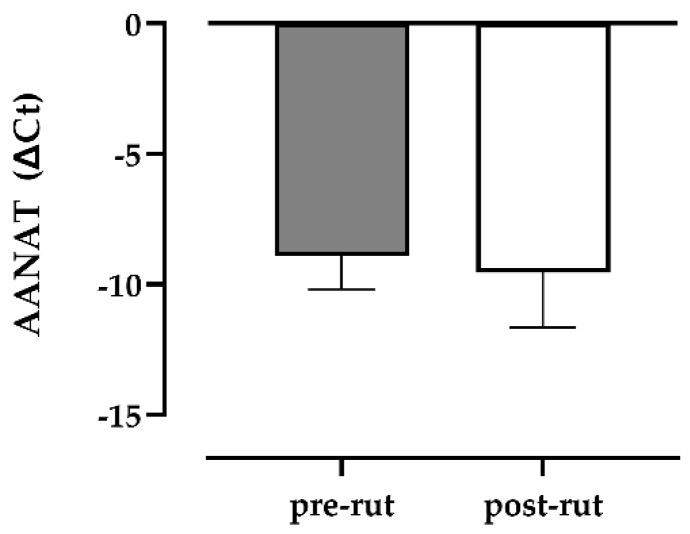
Differences between pre-and post-rut groups for the gene expression of the enzyme AANAT (aralkylamine-N-acetyltransferase).

The specificity of all PCR products was verified in relation to melting curve analysis and agarose gel electrophoresis, and to the positive control (testis from bovine) in which all the analyzed transcripts were detectable. Among the studied genes, only AANAT was detectable in all roe deer samples (*n* = 18), without any difference of expression (Figure 2), whereas ASMT, UCP1 and MT1 were undetectable and MT2 was detectable in only a few samples (3/17).

### 3.3. Western Blot Analysis of MTR1A and MTR1B

**Figure 3:**
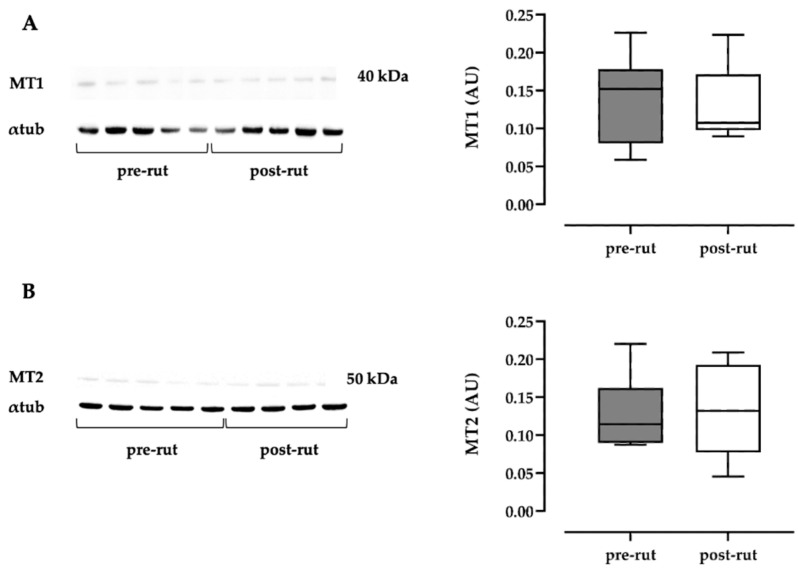
Western blot analyses and comparisons between pre- and post-rut periods for the melatonin receptors 1 (**A**) and 2 (**B**). α tub, alfa-tubulin; MWM, molecular weight markers. (original western blot figures in Figure S1).

MT1 Western blot analysis revealed a band of expected molecular weight (approximately 39 kDa [37,40]) in all samples (Figure 3A); MT2 Western blot analysis revealed a band of expected molecular weight (approximately 42 kDa [37,40]) in all samples (Figure 3B). For both analyses, no significant differences between the two groups were recorded.

### 3.4. Correlation Analysis

**Figure 4:**
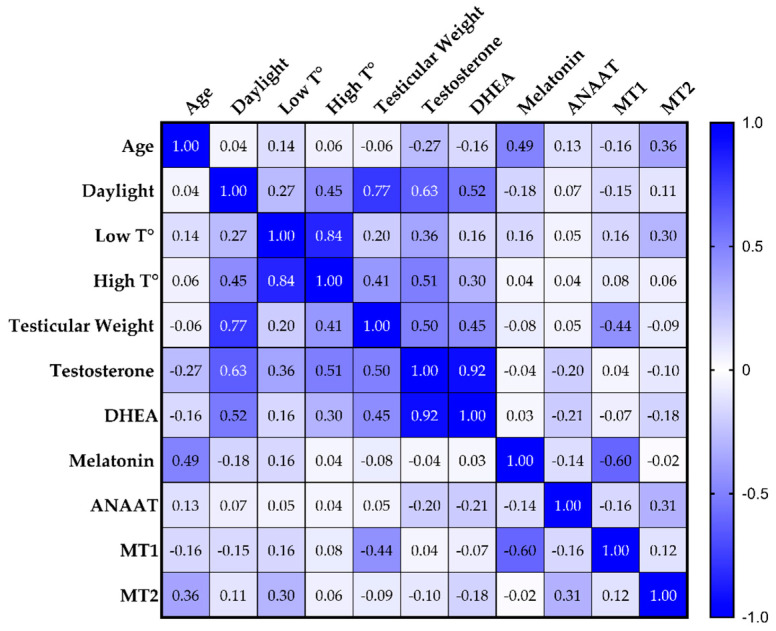
Color-coded Spearman’s rank correlation coefficients (ρ) table. Blue background indicates perfect correlations, white shows that the two variables do not vary together; light blue gradients indicate different levels of correlation. DHEA, dehydroepiandrosterone; T°, temperature; AANAT, aralkylamine-N-acetyltransferase; MT1 and MT2, melatonin receptors 1 and 2.

Along with the statistical correlation between testicular weight and its testosterone level, the Spearman’s correlation table (Figure 4) showed that melatonin was inversely correlated with MT1 (ρ = −0.60; *p* = 0.008). DHEA was correlated with testicular weight (ρ = 0.45; *p* = 0.062) and with testicular testosterone (ρ = 0.92; *p* < 0.0001). Testosterone, DHEA, and testicular weight were correlated with daylight, with ρ = 0.63 (*p* = 0.005), ρ = 0.52 (*p* = 0.026), and ρ = 0.77 (*p* < 0.001), respectively. Testosterone was also correlated with a high environmental temperature (ρ = 0.051; *p* = 0.031). Other correlations were highlighted by the analysis.

## 4. Discussion

This study present new data regarding the physiological behavior of melatonin within the testes of roe deer bucks in two different physiological moments, before and after rutting, including its receptors and synthesis/function pathways. As reported in the literature for this species [25,27,30], the testis shows its functional peak in the pre-rut period (June/mid-July), with high levels of testosterone and increased spermatogenesis in respect to the post-rut period (mid-August/September). Such patterns were confirmed by the authors in a previous publication on the same animals analyzed in the present study [35], and further strengthened by the quantification of DHEA performed in the present project. Indeed, the levels of such testosterone precursors were also statistically higher in the pre-rut when compared the post-rut period. Additionally, DHEA levels were correlated with the testicular weight, mimicking what has already been reported for testosterone in the same animals [35]. The morpho-functional changes between the pre- and post-rut periods were strengthened and confirmed by the Spearman’s rank correlation analysis. Indeed, the decrease in daylight hours relates to a decrease in testicular weight and hormonal levels.

Better investigating the role of melatonin in such seasonal breeders may help to unravel the mechanisms underlying the roe deer testicular cycle. The present study, to the best of the authors’ knowledge, represents the first report regarding the quantification of melatonin in the testicular tissue of roe deer bucks during pre- and post-rut periods, along with the quantification of its receptors and the gene expression analysis of its local pathways of synthesis (AANAT and ASMT) and function (UCP1).

Generally speaking, melatonin is synthesized and secreted by the pineal gland, also known as the *epiphysis cerebri*, although it can also be “locally” synthesized by other tissues/organs such as the gastrointestinal wall [41] and the ovine corpus luteum [42]. Indeed, when examining different mammalian species, the local synthesis of melatonin within the testes has been reported, for example, in rams, rats, and bison [16,21,22,43]. In a hamster model, melatonin was clarified to also have a local role as a steroidogenesis modulator [44]. Accordingly, the melatonin quantified in the testicular tissue of roe deer bucks in the present study may derive from both the pineal gland and the testes.

Discussing the absence of statistical differences between the testicular melatonin levels of animals culled during pre- and post-rut periods can be challenging, because no comparable data are available in the literature. Examining at other matrices, and in particular peripheral blood, a recent study has reported how melatonin seems to be higher in May, with a following drop in summer, and then slowly increases in September [37]. This finding is in contrast with our data which show an overall increasing trend, with high individual variability in the levels on melatonin within the testes during the post-rut period. Such differences between blood and testicular levels of hormones may be indicative of a local production of melatonin within the male gonads, as previously hypothesized. However, it is important to acknowledge that an increase in melatonin in the post-rut period has to be expected, according to its physiology, as daylight hours decrease as summer progresses. Moreover, higher levels of melatonin in the post-rut period may be related to the increased local metabolic activity for tissue remodeling, with higher chances of oxidative stress. It is indeed recognized that melatonin is a powerful antioxidant and free radical scavenger capable of protecting against multiple types of tissue damage; for example, within porcine oocytes [45]. Finally, one of the possible reasons behind the lack of statistical differences for this parameter may be the number of samples available, which was unfortunately relatively low in light of the abovementioned individual variability.

In order to investigate a potential local production of melatonin within the testes, different genes codifying for pivotal proteins in synthesis processes, already reported in different extra-pineal tissues of other species as the brain, retina, gastrointestinal tract, placenta and reproductive tract [21,46], were studied. Out of the analyzed samples, only AANAT was detectable and quantifiable in both groups.

This gene encodes for aralkylamine N-Acetyltransferase, also known as serotonin N-acetyltransferase, the enzyme responsible for the transformation of serotonin into N-Acetyl-serotonin, the final precursor of melatonin [21,22]. No quantitative differences were noted between the two groups, although the presence of this gene may further strengthen the hypothesis of a local synthesis of melatonin within the testes of roe deer bucks. Nonetheless, the relative stability of AANAT gene expression levels is in agreement with the previously discussed absence of significant melatonin fluctuations between the pre- and post-rut periods. In this case, the lack of differences between pre- and post-rut samples is most likely due to the very short sampling timeframe, dictated by the local hunting calendar. Indeed, reports in the literature suggest that, in another “long days seasonal breeder” such as the hamster, an induced short photoperiod led to a significant testicular increase in this gene only after 10 weeks of exposure. The same study also reported a significant increase in testicular melatonin, after 10 weeks, strengthening the hypothesis that the lack of statistical differences reported here, despite a marked increasing trend, may be due to the sampling timeframe [17]. Expanding the samplings to the entire solar year may unveil differences in AANAT gene expression, for example during early spring, when testes start to regain their functional capabilities in preparation for the breeding season. Overall, what can be said regarding gene expression, based on the results of the present study, is that the analyzed enzymes do not seem to play a pivotal role in the regression phase of testicular morphology and physiology.

The two main melatonin receptors, MT1 and MT2, were quantifiable by means of Western blotting, as previously reported [37], but not from an mRNA point of view. The data shown here seem to be in contrast with those already reported [37], but the sampling periods and geographical areas are not comparable, potentially leading to different findings when exploring such “time/location-related” parameters. The absence of protein differences between animals culled during the pre- and post-rut periods suggests a relative stability in their presence within testicular parenchymal samples. This is coherent with the fact that protein quantification was successful, but gene expression was not, suggesting that, during the sampling’s timeframe, the production of MT1 and MT2 was stable with a low level of mRNA expression and not up- or downregulated. As previously stated, expanding the sampling timeframe may also provide additional information in this case as, for example, already reported in the European bison, where MT1 and 2 receptors were higher in December when compared to June [16].

Finally, no correlations were highlighted between melatonin-related parameters and testicular morph-functional aspects (such as testicular weight and hormonal concentrations).

## 5. Conclusions

This study, to the best of the authors’ knowledge, represents the first report regarding the melatonin system in the roe deer testis. None of the investigated parameters related to the melatonin pathway showed statistical differences between the two groups, potentially due to the very short sampling timeframe. Melatonin was quantified, for the first time, within the testicular parenchyma of wild roe deer bucks, along with its two main receptors. More studies are necessary in order to understand the role of this molecule in the peculiar testicular cycle of this species.

## Figures and Tables

**Table 1:** Descriptive statistics for all the investigated parameters.

	Pre-Rut		Post-Rut	
	Mean (SD)	Min; Max	Mean (SD)	Min; Max
Animals				
Age (months) ^§^	39 (15)	24; 72	32 (10)	15; 48
Testis				
Weight (g) ^§^ *	20.47 (5.26)	10.24; 26.61	11.11 (5.26)	7.20; 23.42
Testosterone (pg/mg) ^§^ *	917.39 (676.30)	248.87; 2419.75	281.18 (261.18)	48.22; 848.00
Melatonin (pg/mg)	0.36 (0.13)	0.14; 0.58	0.59 (0.34)	0.23; 1.26
DHEA (pg/mg) *	135.15 (130.00)	17.72; 443.6	36.20 (34.16)	3.1; 86.25
Environmental data				
T min (°C)	15.00 (1.32)	13; 17	12.56 (4.85)	5; 17
T max (°C)	28.89 (2.85)	23; 32	26.44 (4.50)	19; 33
Daylight	13 h 52 min (14 min)	13 h 28 min; 14 h 12 min	10 h 41 min (52 min)	10 h 30 min; 12 h 11 min
Gene expression				
AANAT (ΔCT)	−8.92 (1.27)	−9.91; −5.99	−9.55 (2.11)	−13.05; −7.42
Western blot				
MT1 (AU)	0.136 (0.056)	0.059; 0.226	0.133 (0.047)	0.090; 0.224
MT2 (AU)	0.127 (0.047)	0.087; 0.220	0.129 (0.058)	0.045; 0.209

AU: arbitrary units; ^§^: data previously published by the authors [35]. *, statistical differences between Pre-rut and Post-rut (*p* < 0.05). DHEA, dehydroepiandrosterone; T, temperature; AANAT, aralkylamine-N-acetyltransferase; MT1/2, melatonin receptors 1/2.

## Data Availability

Data are contained within the article or Supplementary Materials. Raw data are available on request from the corresponding author.

## Supplementary Materials

The following are available online at https://www.mdpi.com/article/10.3390/ani11071874/s1, Figure S1: Original western blot figures for Figure 3A,B.

## References

[1] Santiago-Moreno J., Gómez-Brunet A., Toledano-Díaz A., Picazo R., Gonzalez-Bulnes A., López-Sebastián A. (2006). Seasonal Endocrine Changes and Breeding Activity in Mediterranean Wild Ruminants. Reprod. Domest. Anim..

[2] Borah B.K., Renthlei Z., Trivedi A.K. (2019). Seasonality in Terai Tree Frog (Polypedates Teraiensis): Role of Light and Temperature in Regulation of Seasonal Breeding. J. Photochem. Photobiol. B.

[3] Mahmoud I.Y., Licht P. (1997). Seasonal Changes in Gonadal Activity and the Effects of Stress on Reproductive Hormones in the Common Snapping Turtle, Chelydra Serpentina. Gen. Comp. Endocrinol..

[4] Sengupta A., Maitra S.K. (2006). The Pineal Gland, but Not Melatonin, Is Associated with the Termination of Seasonal Testicular Activity in an Annual Reproductive Cycle in Roseringed Parakeet Psittacula Krameri. Chronobiol. Int..

[5] Dawson A. (2015). Annual Gonadal Cycles in Birds: Modeling the Effects of Photoperiod on Seasonal Changes in GnRH-1 Secretion. Front. Neuroendocrinol..

[6] Pastor L.M., Zuasti A., Ferrer C., Bernal-Mañas C.M., Morales E., Beltrán-Frutos E., Seco-Rovira V. (2011). Proliferation and Apoptosis in Aged and Photoregressed Mammalian Seminiferous Epithelium, with Particular Attention to Rodents and Humans. Reprod. Domest. Anim..

[7] Dadhich R.K., Barrionuevo F.J., Real F.M., Lupiañez D.G., Ortega E., Burgos M., Jiménez R. (2013). Identification of Live Germ-Cell Desquamation as a Major Mechanism of Seasonal Testis Regression in Mammals: A Study in the Iberian Mole (Talpa Occidentalis). Biol. Reprod..

[8] Blottner S., Roelants H., Waganer A., Wenzel U.D. (1999). Testicular Mitosis, Meiosis and Apoptosis in Mink (Mustela Vison) during Breeding and Non-Breeding Seasons. Anim. Reprod. Sci..

[9] Tabecka-Lonczynska A., Mytych J., Solek P., Kowalewski M.P., Koziorowski M. (2019). Seasonal Expression of Insulin-like Growth Factor 1 (IGF-1), Its Receptor IGF-1R and Klotho in Testis and Epididymis of the European Bison (Bison Bonasus, Linnaeus 1758). Theriogenology.

[10] viviD D., Bentley G.E. (2018). Seasonal Reproduction in Vertebrates: Melatonin Synthesis, Binding, and Functionality Using Tinbergen’s Four Questions. Molecules.

[11] Wehr T.A. (1997). Melatonin and Seasonal Rhythms. J. Biol. Rhythms.

[12] Frungieri M.B., Calandra R.S., Rossi S.P. (2017). Local Actions of Melatonin in Somatic Cells of the Testis. Int. J. Mol. Sci..

[13] Reiter R.J., Tan D.-X., Manchester L.C., Paredes S.D., Mayo J.C., Sainz R.M. (2009). Melatonin and Reproduction Revisited. Biol. Reprod..

[14] Reiter R.J. (1991). Pineal Melatonin: Cell Biology of Its Synthesis and of Its Physiological Interactions. Endocr. Rev..

[15] Yu K., Deng S.-L., Sun T.-C., Li Y.-Y., Liu Y.-X. (2018). Melatonin Regulates the Synthesis of Steroid Hormones on Male Reproduction: A Review. Molecules.

[16] Tabecka-Lonczynska A., Mytych J., Solek P., Kulpa M., Koziorowski M. (2017). New Insight on the Role of Melatonin Receptors in Reproductive Processes of Seasonal Breeders on the Example of Mature Male European Bison (Bison Bonasus, Linnaeus 1758). J. Photochem. Photobiol. B.

[17] Mukherjee A., Haldar C. (2014). Photoperiodic Regulation of Melatonin Membrane Receptor (MT1R) Expression and Steroidogenesis in Testis of Adult Golden Hamster, Mesocricetus Auratus. J. Photochem. Photobiol. B.

[18] Yang W.-C., Tang K.-Q., Fu C.-Z., Riaz H., Zhang Q., Zan L.-S. (2014). Melatonin Regulates the Development and Function of Bovine Sertoli Cells via Its Receptors MT1 and MT2. Anim. Reprod. Sci..

[19] González-Arto M., Aguilar D., Gaspar-Torrubia E., Gallego M., Carvajal-Serna M., Herrera-Marcos L.V., Serrano-Blesa E., Hamilton T.R.D.S., Pérez-Pé R., Muiño-Blanco T. (2017). Melatonin MT₁ and MT₂ Receptors in the Ram Reproductive Tract. Int. J. Mol. Sci..

[20] Izzo G., Francesco A., Ferrara D., Campitiello M.R., Serino I., Minucci S., d’Istria M. (2010). Expression of Melatonin (MT1, MT2) and Melatonin-Related Receptors in the Adult Rat Testes and during Development. Zygote Camb. Engl..

[21] Gonzalez-Arto M., Hamilton T.R.D.S., Gallego M., Gaspar-Torrubia E., Aguilar D., Serrano-Blesa E., Abecia J.A., Pérez-Pé R., Muiño-Blanco T., Cebrián-Pérez J.A. (2016). Evidence of Melatonin Synthesis in the Ram Reproductive Tract. Andrology.

[22] Tijmes M., Pedraza R., Valladares L. (1996). Melatonin in the Rat Testis: Evidence for Local Synthesis. Steroids.

[23] Frungieri M.B., Gonzalez-Calvar S.I., Rubio M., Ozu M., Lustig L., Calandra R.S. (1999). Serotonin in Golden Hamster Testes: Testicular Levels, Immunolocalization and Role during Sexual Development and Photoperiodic Regression-Recrudescence Transition. Neuroendocrinology.

[24] Frungieri M.B., Zitta K., Pignataro O.P., Gonzalez-Calvar S.I., Calandra R.S. (2002). Interactions between Testicular Serotoninergic, Catecholaminergic, and Corticotropin-Releasing Hormone Systems Modulating CAMP and Testosterone Production in the Golden Hamster. Neuroendocrinology.

[25] Blottner S., Hingst O., Meyer H.H.D. (1996). Seasonal Spermatogenesis and Testosterone Production in Roe Deer (Capreolus Capreolus). J. Reprod. Fertil..

[26] Schams D., Barth D. (1982). Annual Profiles of Reproductive Hormones in Peripheral Plasma of the Male Roe Deer (Capreolus Capreolus). J. Reprod. Fertil..

[27] Roelants H., Schneider F., Göritz F., Streich J., Blottner S. (2002). Seasonal Changes of Spermatogonial Proliferation in Roe Deer, Demonstrated by Flow Cytometric Analysis of c-Kit Receptor, in Relation to Follicle-Stimulating Hormone, Luteinizing Hormone, and Testosterone. Biol. Reprod..

[28] Blottner S., Schön J., Roelants H. (2007). Apoptosis Is Not the Cause of Seasonal Testicular Involution in Roe Deer. Cell Tissue Res..

[29] Schön J., Göritz F., Streich J., Blottner S. (2004). Histological Organization of Roe Deer Testis throughout the Seasonal Cycle: Variable and Constant Components of Tubular and Interstitial Compartment. Anat. Embryol..

[30] Ventrella D., Elmi A., Barone F., Carnevali G., Govoni N., Bacci M.L. (2018). Hair Testosterone and Cortisol Concentrations in Pre- and Post-Rut Roe Deer Bucks: Correlations with Blood Levels and Testicular Morphometric Parameters. Animals.

[31] Schön J., Blottner S. (2008). Estrogens Are Involved in Seasonal Regulation of Spermatogenesis and Sperm Maturation in Roe Deer (Capreolus Capreolus). Gen. Comp. Endocrinol..

[32] Sempéré A.J., Mauget R., Bubenik G.A. (1992). Influence of Photoperiod on the Seasonal Pattern of Secretion of Luteinizing Hormone and Testosterone and on the Antler Cycle in Roe Deer (Capreolus Capreolus). J. Reprod. Fertil..

[33] Kozioł K., Koziorowski M. (2013). Steroid Hormones in Peripheral Blood Plasma and Androgen Receptors in Testis and Epididymis of Roe Deer Male (Capreolus Capreolus) during the Reproduction Season. Small Rumin. Res..

[34] Klonisch T., Schön J., Hombach-Klonisch S., Blottner S. (2006). The Roe Deer as a Model for Studying Seasonal Regulation of Testis Function. Int. J. Androl..

[35] Elmi A., Zannoni A., Govoni N., Bertocchi M., Forni M., Ventrella D., Bacci M.L. (2020). Uncovering the Physiological Mechanisms Underlying the Roe Deer (Capreolus Capreolus) Testicular Cycle: Analyses of Gelatinases and VEGF Patterns and Correlation with Testes Weight and Testosterone. Animals.

[36] Koziorowska-Gilun M., Fraser L., Gilun P., Koziorowski M., Kordan W. (2015). Activity of Antioxidant Enzymes and Their MRNA Expression in Different Reproductive Tract Tissues of the Male Roe Deer (Capreolus Capreolus) during the Pre-Rut and Rut Seasons. Small Rumin. Res..

[37] Kozioł K., Broda D., Romerowicz-Misielak M., Nowak S., Koziorowski M. (2020). Melatonin Concentration in Peripheral Blood and Melatonin Receptors (MT1 and MT2) in the Testis and Epididymis of Male Roe Deer during Active Spermatogenesis. Theriogenology.

[38] Munley K.M., Deyoe J.E., Ren C.C., Demas G.E. (2020). Melatonin Mediates Seasonal Transitions in Aggressive Behavior and Circulating Androgen Profiles in Male Siberian Hamsters. Horm. Behav..

[39] Elmi A., Galligioni V., Govoni N., Bertocchi M., Aniballi C., Bacci M.L., Sánchez-Morgado J.M., Ventrella D. (2020). Quantification of Hair Corticosterone, DHEA and Testosterone as a Potential Tool for Welfare Assessment in Male Laboratory Mice. Anim. Open Access J..

[40] González-Arto M., Vicente-Carrillo A., Martínez-Pastor F., Fernández-Alegre E., Roca J., Miró J., Rigau T., Rodríguez-Gil J.E., Pérez-Pé R., Muiño-Blanco T. (2016). Melatonin Receptors MT1 and MT2 Are Expressed in Spermatozoa from Several Seasonal and Nonseasonal Breeder Species. Theriogenology.

[41] Barajas-López C., Peres A.L., Espinosa-Luna R., Reyes-Vázquez C., Prieto-Gómez B. (1996). Melatonin Modulates Cholinergic Transmission by Blocking Nicotinic Channels in the Guinea-Pig Submucous Plexus. Eur. J. Pharmacol..

[42] Xiao L., Hu J., Zhao X., Song L., Zhang Y., Dong W., Zhang Q., Ma Y., Li F. (2018). Expression of Melatonin and Its Related Synthase and Membrane Receptors in the Oestrous Corpus Luteum and Corpus Luteum Verum of Sheep. Reprod. Domest. Anim. Zuchthyg..

[43] Stefulj J., Hörtner M., Ghosh M., Schauenstein K., Rinner I., Wölfler A., Semmler J., Liebmann P.M. (2001). Gene Expression of the Key Enzymes of Melatonin Synthesis in Extrapineal Tissues of the Rat. J. Pineal Res..

[44] Frungieri M.B., Mayerhofer A., Zitta K., Pignataro O.P., Calandra R.S., Gonzalez-Calvar S.I. (2005). Direct Effect of Melatonin on Syrian Hamster Testes: Melatonin Subtype 1a Receptors, Inhibition of Androgen Production, and Interaction with the Local Corticotropin-Releasing Hormone System. Endocrinology.

[45] Lan M., Zhang Y., Wan X., Pan M.-H., Xu Y., Sun S.-C. (2020). Melatonin Ameliorates Ochratoxin A-Induced Oxidative Stress and Apoptosis in Porcine Oocytes. Environ. Pollut..

[46] Acuña-Castroviejo D., Escames G., Venegas C., Díaz-Casado M.E., Lima-Cabello E., López L.C., Rosales-Corral S., Tan D.-X., Reiter R.J. (2014). Extrapineal Melatonin: Sources, Regulation, and Potential Functions. Cell. Mol. Life Sci. CMLS.

