# Supplementary figures and images for: Testicular Melatonin and Its Pathway in Roe Deer Bucks (Capreolus capreolus) during Pre- and Post-Rut Periods: Correlation with Testicular Involution
Source: Animals (Basel). 2021 Jun 23;11(7):1874. doi: 10.3390/ani11071874 (PMC8300110; doi:10.3390/ani11071874)

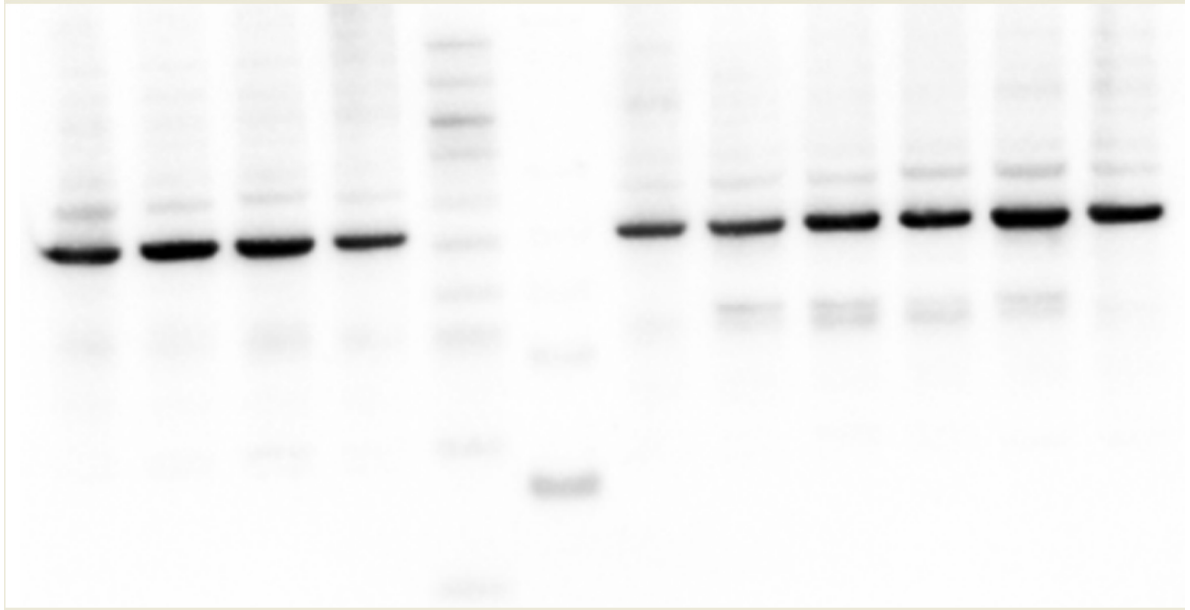

(A) Tub – MT1

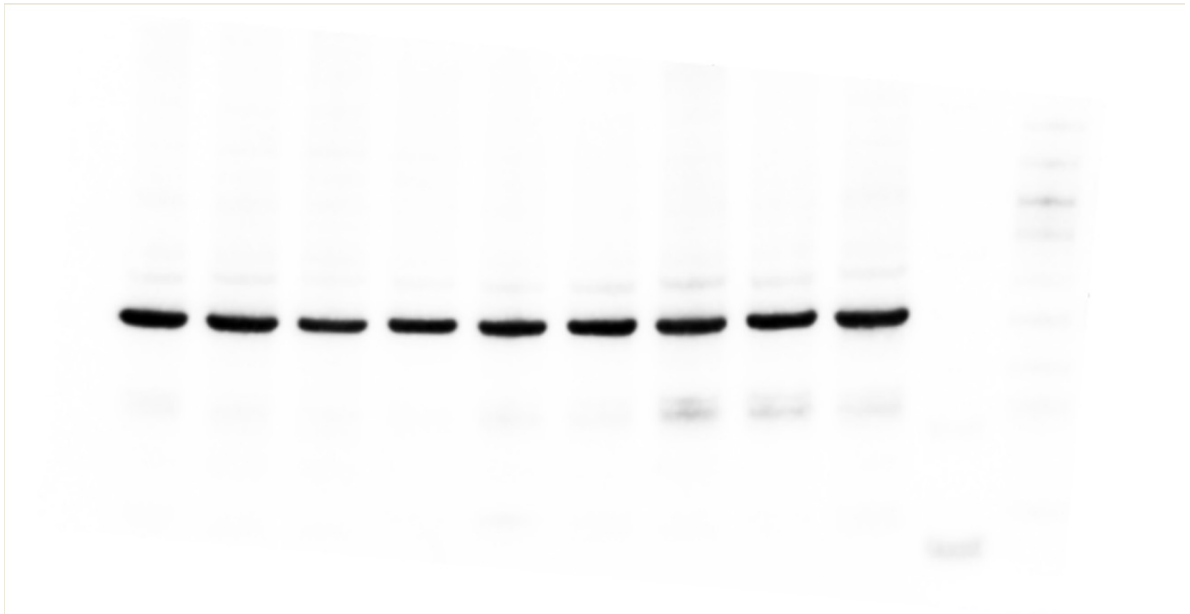

(B) Tub – MT2

**Figure 1.** Original western blot figures. (A) Tub-MT1, (B) Tub-MT2.

Supplement: Supplementary file 1 [file animals-11-01874-s001.zip › animals-1248794-supplementary.pdf]
